# Molar pregnancy with a coexisting living fetus: a case series

**DOI:** 10.1186/s12884-022-05004-3

**Published:** 2022-09-03

**Authors:** Reda Hemida, Eman Khashaba, Khaled Zalata

**Affiliations:** 1 Gynecologic Oncology Unit, Department of Obstetrics and Gynecology, Mansoura University, 35111 Elgomhuria street, Mansoura, Egypt; 2 Department of Community Medicine, Faculty of Medicine, Mansoura University, Mansoura, Egypt; 3 Department of Pathology, Faculty of Medicine, Mansoura University, Mansoura, Egypt

**Keywords:** Molar pregnancy, Living fetus, Outcome

## Abstract

**Background:**

Coexistence of molar pregnancy with living fetus represents a challenge in diagnosis and treatment. The objective of this study to present the outcome of molar pregnancy with a coexisting living fetus who were managed in our University Hospital in the last 5 years.

**Methods:**

We performed a retrospective analysis of patients who presented with molar pregnancy with a coexisting living fetus to our Gestational Trophoblastic Clinic, Mansoura University, Egypt from September, 2015 to August, 2020. Clinical characteristics of the patients, maternal complications as well as fetal outcome were recorded. The patients and their living babies were also followed up at least 6 months after delivery.

**Results:**

Twelve pregnancies were analyzed. The mean maternal age was 26.0 (SD 4.1) years and the median parity was 1.0 (range 0–3). Duration of the pregnancies ranged from 14 to 36 weeks. The median serum hCG was 165,210.0 U/L (range 7662–1,200,000). Three fetuses survived outside the uterus (25%), one of them died after 5 months because of congenital malformations. Histologic diagnosis was available for 10 of 12 cases and revealed complete mole associated with a normal placenta in 6 cases (60%) and partial mole in 4 cases (40%). Maternal complications occurred in 6 cases (50%) with the most common was severe vaginal bleeding in 4 cases (33.3%). There was no significant association between B-hCG levels and maternal complications (*P* = 0.3).

**Conclusion:**

Maternal and fetal outcomes of molar pregnancy with a living fetus are poor. Counseling the patients for termination of pregnancy may be required.

**Trial registration:**

The study was approved by Institutional Research Board (IRB), Faculty of Medicine, Mansoura University (number: R.21.10.1492).

## Introduction

Hydatidiform mole is a rare complication of early pregnancy characterized by disordered proliferation of trophoblastic epithelium and villous edema. It includes complete (CHM) and partial (PHM) hydatidiform moles [1, 2]. Partial hydatidiform mole arises as a result of dispermic fertilization of a haploid oocyte, which produces a triploid set of chromosomes and is commonly associated with congenital fetal malformations [3]. Hydatidiform moles are usually presented with first trimester vaginal bleeding, passage of vesicles, abdominal pain, excessive nausea and vomiting, and rapid abdominal enlargement [1]. Hyperthyroidism and preeclampsia may be present in some cases of complete hydatidiform moles [4, 5]. Human chorionic gonadotropin (hCG) level is elevated but the level in CHM is higher than PHM [6].

Although complete hydatidiform moles can be easily diagnosed using routine ultrasound assessments early in the first trimester by appearance of snow-storm appearance of the placenta; PHM may mimic missed or incomplete abortion [7].

During management of molar pregnancy with a coexisting living fetus; the gynecologist should remind that there are three different types. The most common is twin pregnancy with one normal fetus with a normal placenta and a CHM; the second type is twin pregnancy with a normal fetus and placenta and a PHM; and the third, and most uncommon, is a singleton pregnancy consisting of a normal fetus and a placenta with PM changes [8]. The latter type was reported to occur in 0.005 to 0.01% of all pregnancies [9]. It is sometimes called “Sad Fetus Syndrome” [7]. Pregnancy with a PHM and a normal fetus evolves to a viable fetus in less than 25% of cases [8]. Such pregnancy has little tendency to invade the myometrium and distant metastasis [6].

Coexistence of molar changes with an apparently healthy fetus is unusual in a case of familial recurrent hydatidiform mole (FRHM). It should be differentiated from mesenchymal dysplasia by morphologic features and immunohistochemistry [10].

To the best of our knowledge, there are no available international guidelines for management of molar pregnancy with a living fetus. The available publications are mostly case reports, so the authors prepared this manuscript to present the experience of our University GTD referral clinic in the management and outcome of these rare cases.

## Patients and methods

In this case series; a retrospective analysis of the patients presented with molar pregnancy with a coexisting living fetus to Gestational Trophoblastic Clinic, Mansoura University, Egypt in 5 years (from September, 2015 to August, 2020). The data of the patients were extracted from the computer and paper files. We included all cases above 18 years with diagnosed molar pregnancy with a living fetus based on clinical, ultrasound, and serum human chorionic gonadotropin (hCG) criteria. The patients who refused to give initial permission to use their data in future research where excluded from the study.

Clinical characteristics including age, parity, obstetric history, gestational age, presenting symptoms, serum hCG on initial diagnosis, and family history, were all recorded. Mode of termination of pregnancy (miscarriage, induction of abortion, hysterotomy, vaginal, or caesarean delivery) was also reported.

Maternal complications during pregnancy, labor, and puerperium were described. Fetal outcomes (miscarriage, congenital fetal malformations, prematurity, or normal) were reported. The patients and living babies were followed up at least for 6 months after delivery**.**

The study was approved by Institutional Research Board (IRB), Faculty of Medicine, Mansoura University (number: R.21.10.1492). The excel data and figures are anonymous.

### Statistical analysis

Data entry and analysis was done using SPSS program, version 23.0 (IBM SPSS Statistics for Windows, Armonk, NY: IBM Corp.) was used to analyze the findings. The qualitative data were described in number and percentage. The quantitative data with normal distribution were described in mean and the standard deviation ($\pm$ SD). Discrete variables were summarized in median and range. Contingency coefficient Chi square was used to compare nominal variables. The statistical significance was considered when *P* value was less than 0.05.

## Results

**Fig. 1:**
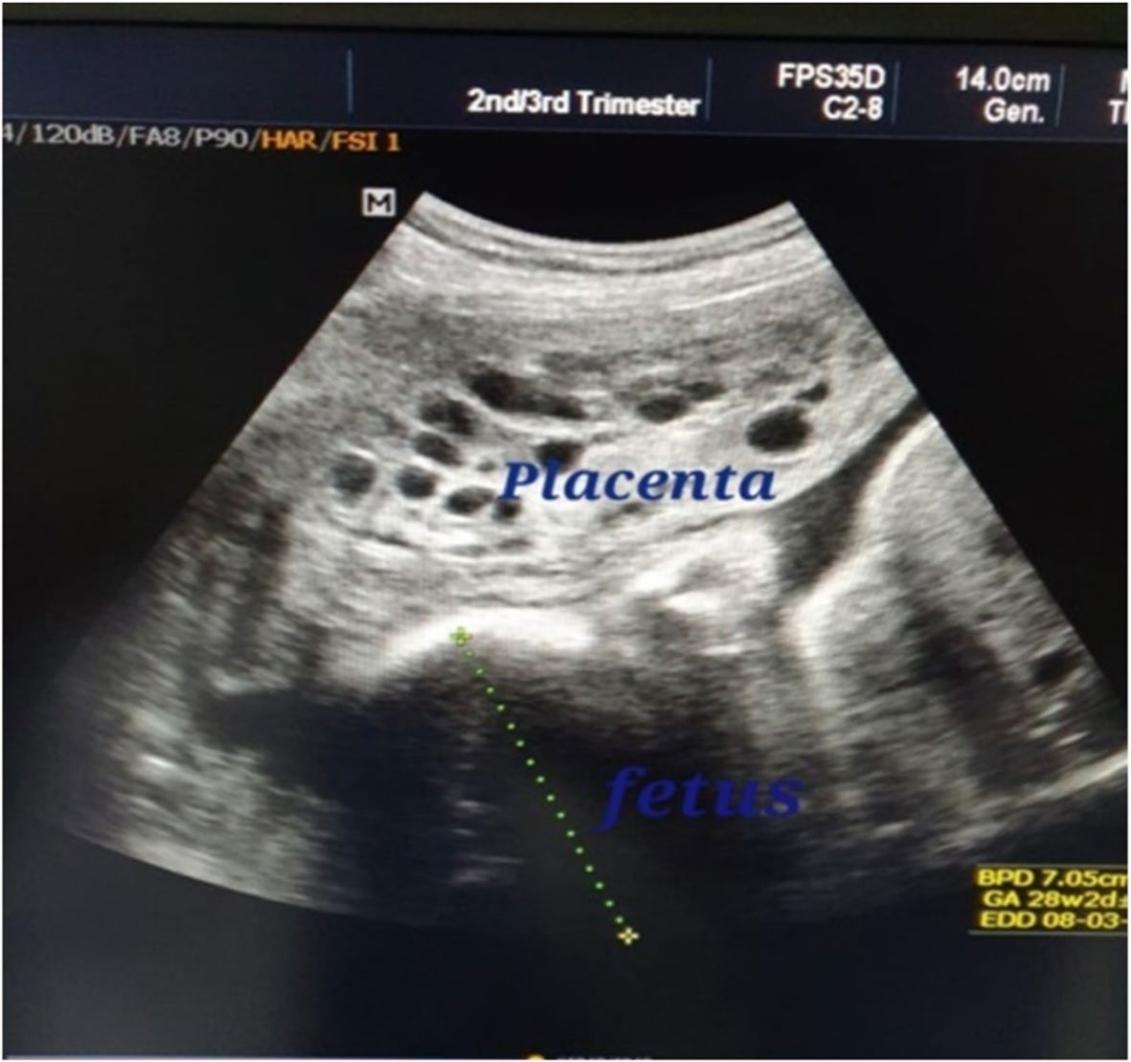
Ultrasound picture of pregnancy of the case (Z) at 28 weeks showing normal fetus with multiple variable-sized vesicles that cannot be separated from another placenta. The fetus is looking morphologically normal

From September 2015 to August, 2020; twelve cases of molar pregnancy with living fetus were managed in our hospital. The mean maternal age was 26.0 ($\pm$ SD 4.1) years while median parity was 1.0 (range 0–3). Duration of pregnancy ranged from 14 to 36 weeks. The median serum hCG at time of diagnosis was 165,210.0 U/L (range 7662–1,200,000). Ultrasound reports showed well-defined multicystic snowstorm-like mass connecting with placenta (Fig. 1). Amniocentesis was performed in one case and revealed a normal diploid female karyotype. During antenatal follow up, the patients who had no complications and requested to undergo conservative treatment were given two injections of 12 mg of betamethasone 24 h apart from 28 weeks of gestation to prevent respiratory distress syndrome.

The fetal outcomes are shown in Table 1; as can be noticed that fetuses survived outside the uterus in three cases (25%). The first two cases were delivered by caesarean delivery at 33 and 36 weeks of gestation after development of persistent abdominal pain and dyspnea with marked abdominal enlargement. Polyhydramnios was excluded by ultrasound examination. The third case delivered vaginally at 36 weeks of a neonate with multiple congenital anomalies namely hydrocephalus and macroglossia who died after 5 months. Seven cases continued their pregnancy beyond 20 weeks; five of them delivered prematurely (71.4%). only one case of them survived after neonatal care admission.

**Table 1:** Maternal complications and fetal outcome among the studied cases

	**Number**	**Percentage**
**Maternal complications:**		
• No complications	6	50%
• Hemorrhage	4	33.3%
• Pre-eclampsia-eclampsia	2	16.7%
• Hyperemesis gravidarum	1	8.3%
• Postmolar GTN	1	8.3%
**Fetal outcome:**		
• Normal, survived	2	16.7%
Delivered at 36 weeks	1	8.3%
Delivered at 33 weeks (needed ICU)	1	8.3%
• CFMF with infantile death^a^	1	8.3%
• Severe prematurity resulted in END**	4	33.4%
• Abortion	5	41.7%
Induced	3	25%
Spontaneous	1	8.3%
Hysterotomy (at 18 weeks)	1	8.3%

^a^CFMF with Infantile death: congenital fetal malformations

Severe prematurity resulted in END**: Two delivered by hysterotomy at 21&22 weeks because of severe bleeding and two spontaneous vaginal deliveries

For the cases who were subjected to cesarean delivery or hysterectomy; the presence of multiple “grape” vesicles on the maternal surface of the placenta was observed.

**Fig. 2:**
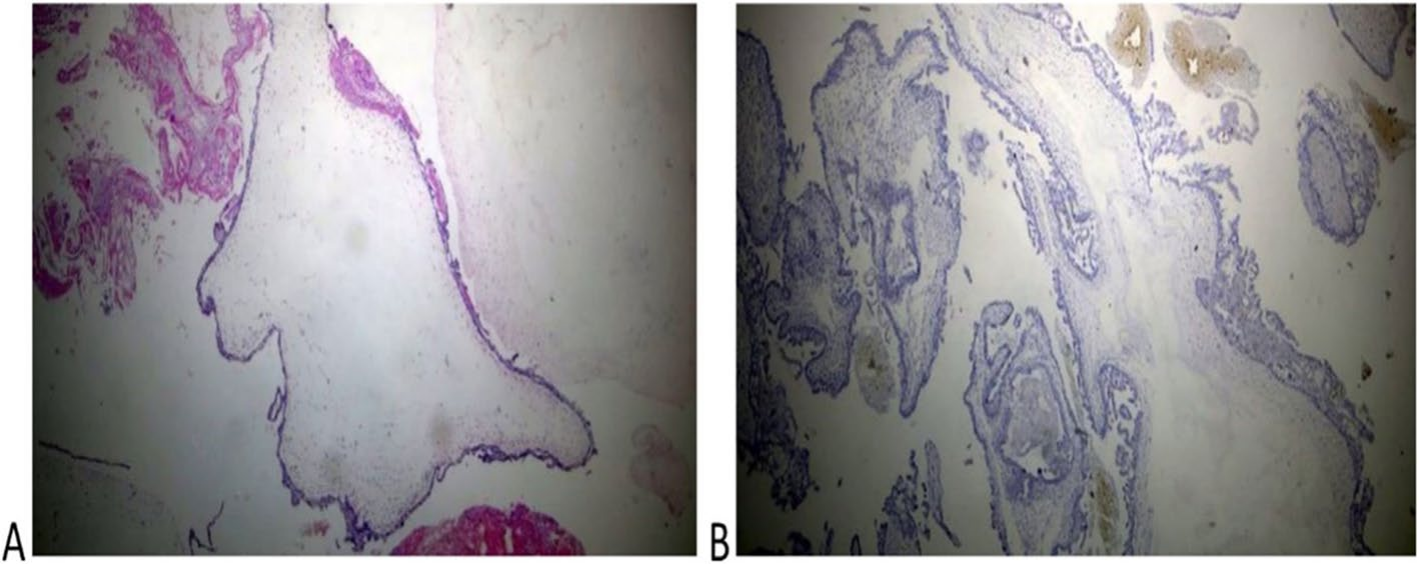
Histopathological examination of the coexistent molar tissues of the case (Z): **A** Complete hydatidiform mole. The picture shows a dilated trophoblastic villous with cistern formation and trophoblastic epithelium hyperplasia (H&E × 100). **B** Complete hydatidiform mole. The picture shows a negative reaction to p57 IHC (Peroxidase × 100)

**Fig. 3:**
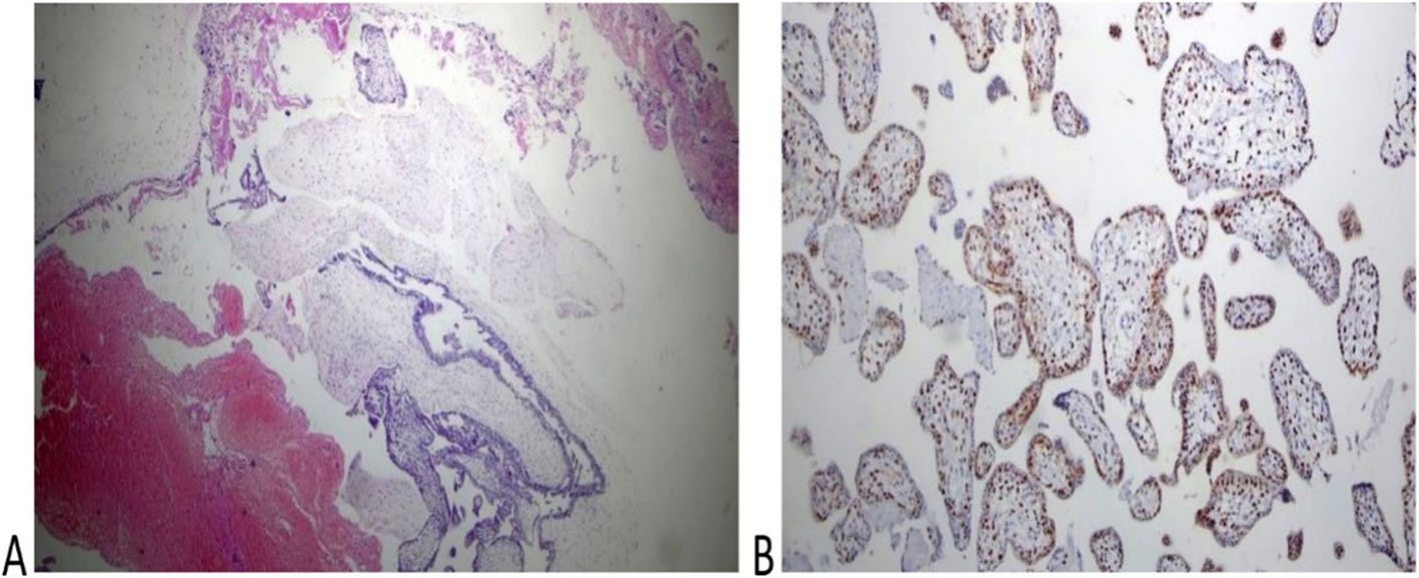
Histopathology of the placenta of the second twin of the case (H): **A** The picture shows normal trophoblastic villi (H&EX100). **B** P57 immunestaining of the same case shows a diffuse positive reaction in both trophoblastic and stromal cells (Peroxidase × 100)

Histologic diagnosis was available for 10 of 12 cases and revealed complete mole associated with a normal placenta in 6 cases (60%) and partial mole in 4 cases (40%)( Figs. 2 and 3). Immunohistochemistry for P57 gene was performed on two cases (Figs. 2 and 3). The first delivered a phenotypically normal alive female baby and its placenta was misdiagnosed as PHM by morphological evaluation but the cytotrophoblast was negative for p57 immunestaining. The second case was diagnosed as dichorionic twins early in pregnancy that was terminated at 14 weeks of gestation because of severe vaginal bleeding. The fetus was phenotypically normal, placenta was histologically normal, and its cytotrophoblast was positive for p57. In addition, there was large amount of molar tissues that was negative for p57 immunostaining demonstrating a diagnosis of a CHM. These data suggest that this conception consists of a dichorionic twins with a living fetus with normal placenta and a CHM (Figs. 2 and 3).

Maternal complications occurred in 6 cases (50%) with the most common was severe uterine bleeding that was observed in 4 cases (33.3%). Other maternal complications are listed in Table 1.

Moreover, three of our patients had familial recurrent hydatidiform mole (FRHM).Two of them are sisters. Genetic study through DNA sequencing confirmed NLRP7 mutations that were previously reported [11]. One of them experienced molar pregnancy with living fetus 3 times when she was aged 25, 27, and 28 years old.

We did not find a significant association between B-hCG level (when considered less than 500,000 and equal or more than 500,000 Unit/liter) and occurrence of maternal complications (*P* = 0.3).

## Discussion

Coexistent molar pregnancy with a living fetus represents a diagnostic and management challenge particularly when the couple is interested to continue pregnancy. In a literature review published by Kawasaki et al. [8]; eighteen cases of molar pregnancies a coexisting living fetus were reported. The mean gestational age at delivery was 24.5 weeks, and only four fetuses could survive outside the uterus (22.2%) indicating a poor fetal outcome. On karyotyping; placenta was diploid in ten cases, indicating that they may be a CHM in a twin pregnancy or associated placental mesenchymal dysplasia that was also reported by Hojberg et al. [12].

The patients with molar pregnancy with coexistent living fetus who were managed in our university hospital in the last 5 years were presented in this report. Among the 12 reviewed pregnancies; three fetuses survived outside the uterus (25%). However, one of them died after 5 months because of congenital malformations that were reported by other authors [3]. The overall fetal survival in our series is less than reported in the literature review [8]. Moreover, Giorgione et al. [13] reported that overall neonatal survival in their series was 45% (5 of 11); the difference may be related to different patient criteria and neonatal care facilities in different hospitals. Among seven pregnancies continued beyond 20 weeks; five ended in premature deliveries (71.4%), which is much higher than the reported global incidence of prematurity allover pregnancies of 11% [14].

Amniocentesis is recommended for cases undergoing conservative treatment to exclude chromosomal abnormalities [15], however, it was performed only in one case in our series. Nine ladies refused the procedure for fear of complications while early pregnancy termination before time of amniocentesis was performed for two patients. In other series [13], prenatal invasive procedures were performed in 8 of 13 cases (62%). The acceptability of the pregnant ladies to perform prenatal invasive procedures differs from a community to another.

We reported occurrence of maternal complications in 50% of the studied cases; the commonest was severe vaginal bleeding. Although Sánchez-Ferrer et al. [15] concluded that termination of pregnancy is not indicated if the fetus is normal and continuation to birth is possible in nearly 60% of cases with no increase in maternal risks when the patient is closely monitored after birth until B-hCG is negative. The difference may be due to different number of cases in each study.

Moreover, two of the managed cases (16.7%) were complicated with early-onset preeclampsia and subsequently the pregnancy was terminated at 22 and 14 weeks of gestation. This finding was also reported by Kawasaki et al. [8]. In our series, we observed one case of complete mole that progressed to GTN (8.3%) and was successfully treated with single-agent chemotherapy, which is similar to a previous case scenario reported by Peng et al. [16].

The diagnostic challenge of a case of molar pregnancy with a coexisting living fetus is to differentiate two different conditions; singleton conception with a partial mole and dizygotic twins consisting of normal fetus with a complete mole. If the ultrasound picture of a normal fetus of an appropriate size for its gestational age together with an abnormal cystic placenta, a twin pregnancy consisting of a normal fetus and a CHM should be suspected [17]. Shaaban et al. [18] suggested that the peculiar “twin peak” sign in ultrasound, in which chorionic tissues extend into the inter-twin membrane, forming a triangular echogenic structure that intervenes the normal twin sac and the molar pregnancy, confirming the presence of a dichorionic twin gestation.

P57 immunestaining was performed in 2 cases of molar pregnancy with apparently normal fetus (Figs. 2 and 3) that confirmed these cases had a dichorionic twin pregnancy consisting of a complete mole with a co-twin of normal fetus and placenta. However, these cases may have been misdiagnosed as partial mole especially when the first ultrasound was done late in pregnancy. Other authors also reported cases of term deliveries of a complete hydatidiform mole with a coexisting living fetus [16, 19, 20].

We did not find a significant association between B-hCG level (when considered less than 500,000 and equal or more than 500,000 Unit/liter) and occurrence of maternal complications (*P* = 0.3). This finding was in agree with Chale-Matsau et al. [21] who concluded that the β-hCG levels do not always correlate with disease severity and prognosis in patients with GTD.

With respect to the reported maternal and fetal complications in our study and other reports, it is necessary to fully inform the pregnant woman of the possible maternal and fetal complications, such as preeclampsia, hyperthyroidism, vaginal bleeding, and theca lutein ovarian cysts. The probability of postpartum development into persistent trophoblastic disease is also high.

The limitations of this study are its retrospective design, limited number of cases, and availability immunohistochemical study of only two cases.

## Conclusion

Maternal and fetal outcome of molar pregnancy with a living fetus is poor. The incidence of prematurity is high (71.4%). Counseling of the patients for termination of pregnancy may be need. A global guideline for management is required.

## Data Availability

Original data and materials are available on request after contacting the corresponding author.

## Abbreviations

GTD: Gestational trophoblastic neoplasia
CHM: Complete hydatidiform mole
PHM: Partial hydatidiform mole
B-hCG: B subunit of human chorionic gonadotropin
GTN: Gestational trophoblastic neoplasia
SD: Standard deviation
